# Cold atmospheric plasma for implant surface decontamination and osseointegration: a scoping review with implications for peri-implantitis management

**DOI:** 10.1186/s12903-026-09164-y

**Published:** 2026-07-17

**Authors:** Kiana Shahraki, Mitra Azizan Bidabadi, Ningbo Zhao

**Affiliations:** 1 Key Laboratory of Shaanxi Province for Craniofacial Precision Medicine Research, College of Stomatology, Xi’an Jiaotong University, Xi’an, China; 2 Clinical Research Center of Shaanxi Province for Dental and Maxillofacial Disease, College of Stomatology, Xi’an Jiaotong University, Xi’an, China; 3 Department of Advanced Implant Dentistry, College of Stomatology, Xi’an Jiaotong University, Xi’an, China; 4 Department of Oral Surgery and Stomatology, School of Dental Medicine, University of Bern, Bern, Switzerland

**Keywords:** Cold atmospheric plasma, Dental implant, Peri-implantitis, Decontamination, Osseointegration

## Abstract

**Background:**

Peri-implantitis is a biofilm-associated inflammatory condition leading to progressive bone loss around dental implants. Effective management requires both implant surface decontamination and re-establishment of osseointegration. Cold atmospheric plasma (CAP) has emerged as a promising approach due to its antimicrobial activity and its ability to modify implant surface properties.

**Methods:**

This scoping review was conducted in accordance with the PRISMA-ScR guidelines. A comprehensive search of PubMed, Scopus, Web of Science, and Google Scholar was performed to identify relevant studies. In vitro, ex vivo, and animal studies investigating the effects of CAP on implant surface decontamination and/or osseointegration were included. Given the limited availability of standardized peri-implantitis models, studies reflecting key biological components of the disease process were considered.

**Results:**

A total of 77 studies were included. Among them, 31 focused on implant surface decontamination and 46 evaluated osseointegration-related outcomes. The included studies were highly heterogeneous in terms of plasma devices, gas composition, exposure parameters, implant materials, and biological models. Overall, CAP demonstrated consistent antibacterial effects and was associated with improved surface hydrophilicity, enhanced osteoblast response, and increased bone-to-implant contact in preclinical settings.

**Conclusions:**

CAP appears to possess a dual potential to support implant surface decontamination and osseointegration-related processes, targeting biological processes relevant to peri-implantitis. However, the evidence is largely preclinical and derived from heterogeneous models that do not uniformly reproduce the clinical condition of peri-implantitis. Therefore, current findings support the biological plausibility of CAP as an adjunctive strategy, while its clinical effectiveness and optimal application protocols remain to be established through well-designed in vivo studies and clinical trials.

**Supplementary Information:**

The online version contains supplementary material available at https://doi.org/10.1186/s12903-026-09164-y.

## Introduction

Since the introduction of the concept of osseointegration in the 1960 s, dental implant therapy has become a widely accepted treatment modality for the rehabilitation of edentulous and partially edentulous patients [1]. Despite its high long-term success rates, peri-implantitis remains a serious biological complication and a significant clinical challenge [2]. Peri-implantitis is a plaque-associated disease affecting the tissues surrounding dental implants, characterized by inflammation of the peri-implant mucosa and progressive loss of supporting bone, which may ultimately result in implant failure [3]. Its primary etiology involves the accumulation of bacterial biofilms on implant surfaces, triggering a host inflammatory response that leads to tissue destruction over time. This pathological process may progress from peri-implant mucositis to peri-implantitis, thereby compromising osseointegration and increasing the risk of implant loss [4]. Clinically, peri-implantitis is characterized by peri-implant pocket formation, suppuration, and progressive bone loss [5]. The reported prevalence ranges from approximately 8% to 25%, depending on the unit of analysis (implant vs. patient level) and population characteristics. A large meta-analysis reported weighted mean prevalences of 25% at the patient level and 18% at the implant level, highlighting the substantial clinical burden of this condition [6–8]. Therefore, the development of effective therapeutic strategies for peri-implantitis remains of critical importance.

The management of peri-implantitis primarily focuses on two key aspects: implant surface decontamination and the re-establishment of osseointegration. Osseointegration, defined as the direct structural and functional connection between living bone and the implant surface, is essential for the long-term success of dental implants [9]. Effective decontamination is critical for removing bacterial biofilms and halting disease progression [10–12]. These two processes are closely interrelated, as successful decontamination is a prerequisite for achieving and maintaining osseointegration. The persistence of biofilms can impair osteoblast adhesion and proliferation, thereby compromising bone regeneration at the implant interface and predisposing to further implant failure [13]. Conventional approaches to implant surface decontamination include mechanical, chemical, and physical methods; however, none of these techniques can completely eliminate biofilms from the complex micro-structured surfaces of implants [12]. Mechanical methods, such as air-powder abrasion and titanium brushes, can partially disrupt biofilms but are often insufficient in intricate regions such as implant threads. Chemical agents, including hydrogen peroxide, citric acid, and photodynamic therapy, show limited efficacy and may induce surface alterations or cytotoxic effects [11, 14]. Physical approaches, such as laser irradiation and waterjet systems, have also been explored; however, their effectiveness depends on implant characteristics and operator factors, limiting their clinical predictability [15].

Cold atmospheric plasma (CAP) has emerged as a promising adjunctive therapy for peri-implantitis. It combines antimicrobial activity, surface modification capability, and potential to enhance tissue healing, making it an attractive therapeutic approach [16]. CAP has been shown to disrupt bacterial membranes and metabolic activity, effectively removing biofilms from implant surfaces [17, 18]. In addition, it can increase implant surface hydrophilicity, thereby promoting cell adhesion and osteogenic activity [19, 20]. By simultaneously inhibiting bacterial colonization and supporting biological processes related to bone regeneration, CAP may represent a promising adjunctive approach for peri-implantitis management.

Previous studies have primarily investigated the effects of CAP on either implant surface decontamination or osseointegration. However, peri-implantitis involves both biofilm-associated infection and impaired bone integration, and an integrated synthesis of these aspects remains limited. Therefore, this scoping review aimed to evaluate the effects of CAP on implant surface decontamination and osseointegration-related outcomes, and to assess their implications for peri-implantitis management. By integrating evidence across different models, this review provides an overview of the biological effects of CAP rather than direct clinical recommendations.

## Materials and methods

### Study protocol

This scoping review was conducted in accordance with the Preferred Reporting Items for Systematic Reviews and Meta-Analyses Extension for Scoping Reviews (PRISMA-ScR) guidelines [21]. The completed PRISMA-ScR checklist has been provided as Supplementary File 1. A predefined study protocol specifying the research question, the inclusion and exclusion criteria, and analytical framework was established prior to the review to ensure methodological transparency and consistency.

## Eligibility criteria

This review was structured based on the Population, Concept, Context (PCC) framework.

*Population*: Implant surfaces affected by peri-implantitis biofilms, osteoblasts, mesenchymal stem cells.

*Concept*: Use of CAP as a treatment modality.

*Context*: Preclinical (in vitro, ex vivo) and animal studies evaluating CAP for decontamination or osseointegration.

The research question was: What are the reported effects of CAP on dental implant surface decontamination and osseointegration-related outcomes in preclinical and animal models relevant to peri-implantitis?

The inclusion criteria, guided by the PCC framework, were as follows:

- In vitro, ex vivo, or in vivo animal studies.
- Studies investigating the use of CAP for dental implant surface decontamination and/or osseointegration.
- Studies involving titanium or zirconia implant materials.
- Quantitative outcomes including Log₁₀ colony-forming units (CFU) reduction, contact angle measurements, osseointegration parameters (bone-to-implant contact, BIC, %), bone area fraction occupancy (BAFO, %), alkaline phosphatase activity, cell adhesion, etc.
- Full-text available in English.

The exclusion criteria were:

- Case reports, review articles, editorials, and conference abstracts.
- Studies using thermal plasma or other unrelated physical treatments.
- Studies do not involve titanium or zirconia implant materials.

### Information sources and search strategy

A comprehensive electronic literature search was conducted in PubMed, Scopus, Web of Science, and Google Scholar to identify studies published up to July 2025. The search strategy combined medical subject headings and free-text terms related to CAP, peri-implant diseases, and implant-related outcomes. The following keywords were used in various combinations: (“cold atmospheric plasma” OR “CAP”) AND (“peri-implantitis” OR “dental implant”) AND (“decontamination” OR “osseointegration”). Detailed search strategies for each database are provided in Supplementary File S2. In addition, the reference lists of relevant review articles and included studies were manually screened to identify additional eligible studies.

### Selection of sources of evidence

Two independent authors (K.S. and M.A.) screened titles and abstracts. Disagreements were resolved through discussion with a third author (N.Z.), and consensus was subsequently reached. Full texts of eligible studies were retrieved, and data were extracted by two independent authors (K.S. and M.A.) using a standardized table. The table included details such as study type (in vitro, ex vivo, in vivo), authors, sample size, material (e.g., titanium, zirconia), surface characteristics, biological species, treatment duration, power settings, and application distance.

### Outcome evaluation

The primary outcomes were defined as (1) decontamination efficacy and (2) osseointegration-related parameters. Decontamination efficacy was assessed using both quantitative and qualitative indicators, including log₁₀ reduction in bacterial CFU, changes in biofilm morphology and structure, and the quantification of residual organic contaminants on treated implant surfaces. Osseointegration-related outcomes included BIC, BAFO, surface hydrophilicity (assessed by water contact angle measurements), and cellular responses such as osteoblast adhesion, proliferation, and alkaline phosphatase activity. The secondary outcomes included the effects of CAP treatment parameters, such as exposure duration, power output, treatment distance, and plasma type, on these primary outcomes.

## Results

**Fig. 1:**
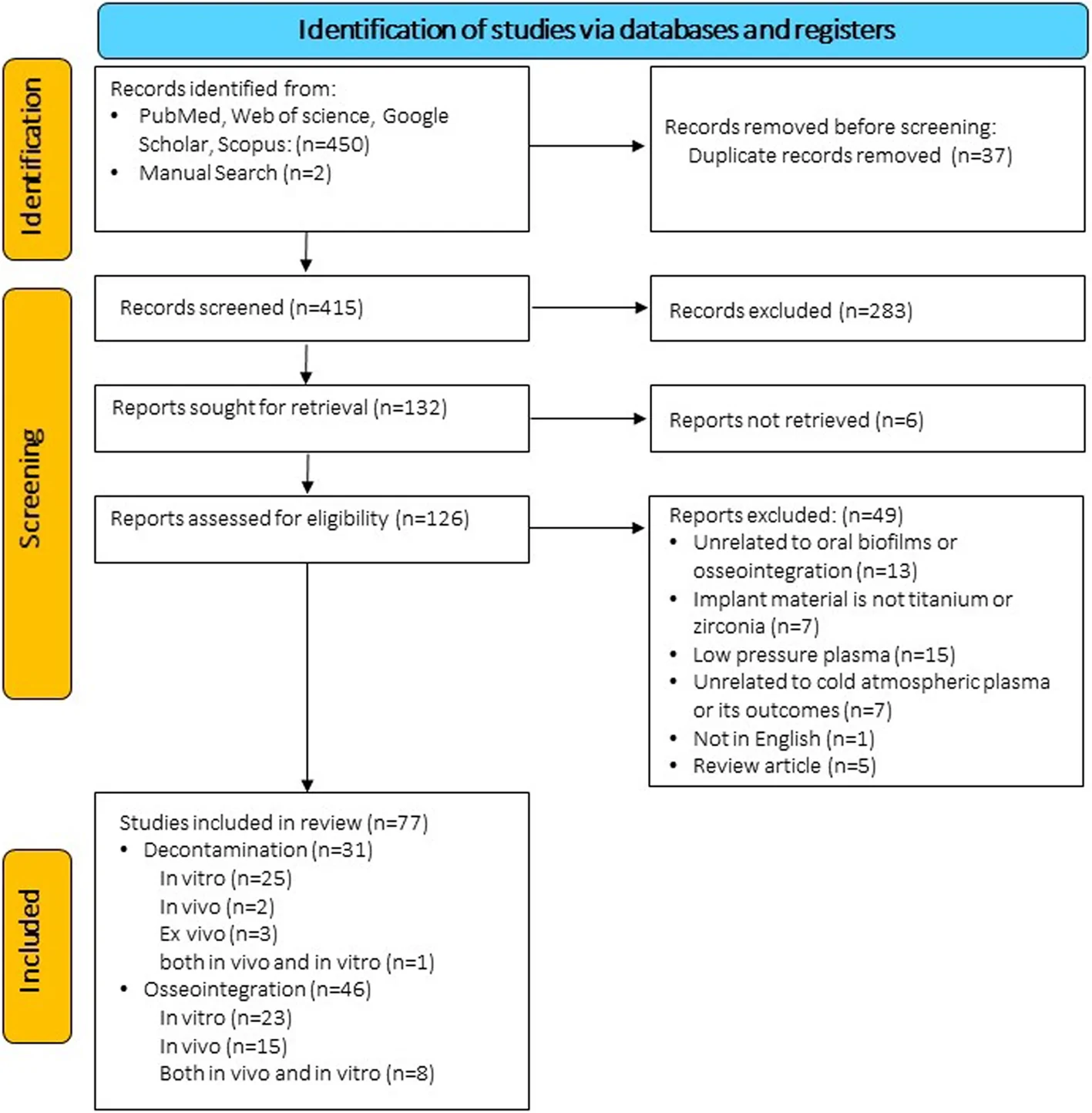
The PRISMA-ScR flowchart of the scoping review

The study selection process is illustrated in Fig. 1. A comprehensive electronic and manual search initially identified 452 records related to the effects of CAP on osseointegration-related outcomes and implant surface decontamination. After removal of 37 duplicates, 415 records remained for screening. Following title and abstract screening and full-text assessment, a total of 77 studies were included for the final analysis.

A total of 31 studies investigating the effects of CAP on implant surface decontamination were included in this review. The characteristics of the included studies are summarized in Table 1. Among the included studies, 25 were conducted in vitro, three were conducted ex vivo, two were conducted in vivo, and one employed both in vitro and in vivo experimental approaches.

**Table 1:** Characteristics of studies on CAP for implant surface decontamination

Study type	Authors	Study model	Total sample size	Surface characteristics	Host species	Biological species	CAP settings			
							**Time**	**Power**	**Gas**	**Distance**
In vivo	Altenburger, M.J., et al. (2025) [22]	Titanium disks	73	Sandblasted and acid-etched	Human participants	Natural mature oral biofilm (mixed oral microbiota)	180 s	NR	Helium	NR
	Zhou, X., et al. (2022) [23]	Titanium implants	20	NR	Beagle dogs	Not specified	180 s	NR	Helium with 0.1% O_2_	≤ 5 mm
In vitro	Yang, Y., et al. (2020) [24]	Zirconia disks	48	Polished/smooth	-	*Streptococcus mutans, Porphyromonas gingivalis*	30, 60 and 90 s	NR	Helium	10 mm
	Flörke, C., et al. (2022) [25]	Titanium implants	45	Sandblasted and acid-etched	-	*Enterococcus faecalis* (ATCC 29, 212)	3 min	5 W	Argon	NR
	Kamionka, J., et al. (2022) [26]	Titanium disks	140	Sandblasted and acid etched and anodized	-	Human subgingival plaque (type of bacteria not specified)	9 min	~ 3.5 W	Argon	5 mm
	Matthes, R., et al. (2022) [27]	Titanium disks	105	Sandblasted and acid-etched	-	Human subgingival plaque (type of bacteria not specified)	140 s	1.6 W	Argon	5 mm
	Hui, W.L., et al. (2021) [28]	Titanium implants	35	Machined and rough alumina particles-blasted	-	Human saliva collected from a patient with peri-implantitis (type of bacteria not specified)	NR	NR	NR	5 mm
	Lee, J.-Y., et al. (2019) [29]	Titanium disks	27	Sandblasted and acid-etched	-	*Porphyromonas gingivalis* (ATCC 33277)	0, 3 and 5 min	NR	Helium	30 mm
	Carreiro, A.F., et al. (2019) [30]	Titanium disks	30	Sandblasted and acid-etched	-	*Porphyromonas gingivalis W83*	1 and 3 min	8 W	Argon	7 mm
	Preissner, S., et al. (2016) [31]	Titanium implants	32	Sandblasted and acid-etched	-	*Streptococcus mitis* (DSM strain 12,643)	60 and 120 s	NR	Argon	8 mm
	Koban, I., et al. (2010) [32]	Titanium disks	320	NR	-	*Candida albicans* (ATCC 10231)	1, 2, 5 and 10 min	3.5 W	Argon or argon with 1% O₂	7 mm
	Ulu, M., et al. (2018) [33]	Titanium disks	76	Sandblasted with large grits and acid-etched	-	*Staphylococcus aureus* (ATCC 25923)	120 s	5 W	NR	1 mm
	Matthes, R., et al. (2023) [15]	Titanium implants and titanium disks	144 implants and 180 disks	Micro-rough titanium implant Sandblasted and acid-etched disks	-	Subgingival plaque from a periodontally diseased volunteer (type of bacteria not specified)	120 s for implants 116 s for disks	1.6 W	Argon	NR
	Hui, W.L., et al. (2021) [13]	Titanium implants	35	Moderately rough, blasted with alumina particles	-	Human salivary biofilm from a peri-implantitis patient (bacterial species not specified)	NR	NR	NR	5 mm
	Lee, C.-M., et al. (2023) [34]	Titanium disks	NR	Sandblasted with large grits and acid-etched	-	*Porphyromonas gingivalis* (KCOM 2796)	30, 60, 90, 120 and 180 s	3.498, 6.387, 6.994 W	Argon	5 mm
	Jungbauer, G., et al. (2022) [35]	Titanium disks	NR	Sandblasted and acid-etched	-	Early colonizers: *Streptococcus gordonii, Actinomyces naeslundii* Bridge bacteria: *Capnocytophaga gingivalis, Campylobacter rectus, Eikenella corrodens, Fusobacterium nucleatum, Prevotella intermedia, Parvimonas micra* Late colonizers: *Porphyromonas gingivalis, Filifactor alocis, Tannerella forsythia, Treponema denticola*	For planktonic bacteria: 10, 30, 60 and 120 s For biofilms: 30, 60 and 120 s	~ 8 W	Ambient air	2 mm
	Lee, J.-H., et al. (2017) [36]	Titanium disks	NR	Roughened	-	*Streptococcus sanguinis* (ATCC 10556)	10 s	NR	Compressed air, amonia and nitrogen	3 mm
	Lee, M.-J., et al. (2019) [37]	Titanium disk	NR	Mirror-polished	-	Gram-positive bacteria: *Streptococcus mutans* and *Staphylococcus aureus* Gram-negative bacteria: *Klebsiella oxytoca* and *Klebsiella pneumoniae*	2 and 10 min	NR	Compressed air	3 mm
	Matthes, R., et al. (2024) [38]	Titanium implants	88	Roughened	-	Artificial, water-insoluble plaque (type of bacteria not specified)	120 s	1.6 W	Argon	NR
	Duske, K., et al. (2015) [39]	Titanium disks	80	Sandblasted and acid-etched	-	Human subgingival plaque biofilm/mixed oral biofilm	18 min	2–3 W	Argon with 1% O₂	5 mm
	Yang, Y., et al. (2021) [17]	Zirconia disks	NR	Smooth/polished	-	*Streptococcus mutans* (UA159 strain)	60 s	NR	Helium	10 mm
	Yang, Y., et al. (2018) [40]	Titanium disks	NR	Mechanically polished	-	*Porphyromonas gingivalis* (ATCC 33277)	2, 4 and 6 min	NR	Air	15 mm
	Haude, S., et al. (2024) [41]	Titanium disks	140	TiUnite® anodized	-	Subgingival plaque from a human volunteer (type of bacteria not specified)	18 min	NR	Argon	5 mm
	Lee, S.-K., et al. (2022) [42]	Zirconia disks	142	Polished/smooth	-	*Porphyromonas gingivalis* cell line: MC3T3-E1	60, 300 and 600 s	300 W	Argon with 0.8% O₂	NR
	Miura, T., et al. (2017) [43]	Titanium disks	NR	Sandblasted and acid-etched	-	*Porphyromonas gingivalis*	10 and 20 s	NR	NR	6 and 8 mm
	Zipprich, H., et al. (2022) [44]	Titanium implants	60	Sandblasted large-grit acid-etched	-	Human saliva (type of bacteria not specified)	6 min	NR	Argon	2 mm
	Panariello, B.H.D., et al. (2022) [18]	Titanium disks	216	NR	-	*Actinomyces naeslundii* ATCC 12104, *Porphyromonas gingivalis* W83, *Streptococcus oralis* ATCC 35037, *Veillonella dispar* ATCC 17748	1, 3 and 5 min	NR	Argon	3 and 10 mm
Ex vivo	Rupf, S., et al. (2011) [45]	Titanium disks	334	Sandblasted and acid-etched	Human participants (2 volunteers)	Multispecies human oral biofilms (type of bacteria not specified)	NR	3 and 5 W	Helium	2 mm
	Idlibi, A.N., et al. (2013) [46]	Titanium disks	200	Smooth/machined	Human participants (5 healthy volunteers)	Natural oral biofilm/mixed oral microorganism	CAP1: 196.25 s CAP2: 196.25 s CAP3: 49.06 s CAP4: 49.06 s	CAP1: 5 W CAP2: 3 W CAP3: 5 W CAP4: 5 W	CAP1: Helium CAP2: Helium CAP3: Helium CAP4: Helium with O₂	2 mm
	Preissner, S., et al. (2019) [47]	Titanium disks	40	Sandblasted and acid-etched	Human participants: (8 healthy volunteers)	Human multispecies oral biofilm (type of bacteria not specified)	90 s	CAP I: NR; CAP II: 450 mW; CAP III: 5 W	CAP I: Argon; CAP II: Ambient air; CAP III: Helium with NO	CAP I: ~ 3 mm; CAP II: 2 mm; CAP III: 3 mm
Combination of in vitro and in vivo	Zeng, Y., et al. (2020) [48]	Titanium implants and titanium disks	NR	Titanate layer with nanonetwork structures	Sprague–Dawley rats	*Staphylococcus aureus*	30 s	NR	NR	5 mm

The 31 included studies showed substantial heterogeneity in implant materials, surface characteristics, and biofilm models. Most studies investigated titanium implants or discs (27/31), whereas only three evaluated zirconia surfaces and one examined titanium modified with titanate nanonetwork structures [17, 24, 42, 48]. Sandblasted and acid-etched titanium was the most frequently studied surface type [15, 22, 25–28, 30, 31, 33–35, 39, 43], while machined, polished, roughened, anodized, and zirconia surfaces were less commonly investigated [13, 17, 24, 28, 36–38, 40–42, 46, 48]. Human-derived oral biofilms were used in 12 studies [13, 15, 22, 26–28, 39, 41, 44–47], whereas 8 studies evaluated single-species biofilms, most commonly *porphyromonas gingivalis* [24, 29, 30, 34, 35, 40, 42, 43]. Only two studies employed defined multispecies peri-implant biofilm models representative of clinical conditions [18, 35].

CAP treatment protocols varied considerably across studies. Exposure times ranged from 10 s to 18 min, although treatment durations of approximately 60–180 s were most common [15, 17, 18, 22, 23, 25, 27, 30, 31, 33–35, 37, 38, 40, 42, 47]. Argon was the predominant working gas [15, 18, 25–27, 30, 31, 34, 38, 41, 44], whereas helium [17, 22, 24, 29, 45] and O_2_-enriched plasma systems [23, 32, 39, 42, 46] were each evaluated in five studies. The treatment distance ranged from 1 to 30 mm, with 2–5 mm being most frequently reported [13, 23, 26–28, 34–37, 39, 41, 44–48]. Overall, current evidence was primarily derived from studies using sandblasted and acid-etched titanium surfaces, argon- or helium-based CAP systems, and human-derived oral biofilms, while data on zirconia implants, standardized treatment protocols, and polymicrobial peri-implantitis biofilms were limited.

A total of 46 studies investigating the effects of CAP on osseointegration-related outcomes were included in this review. The characteristics of the included studies are summarized in Table 2. The included studies demonstrated substantial heterogeneity in study design, implant materials, surface characteristics, and CAP protocols. Fifteen studies were conducted in vivo [20, 23, 49–61], 23 were performed in vitro [19, 36, 39, 40, 42, 62–79], and eight combined both in vitro and in vivo approaches [48, 80–86]. Titanium was the predominant substrate and was investigated in 40 studies, whereas zirconia was evaluated in only six studies [42, 50, 55, 64, 70, 75]. The most frequently studied surfaces were sandblasted and acid-etched or related roughened titanium surfaces [20, 39, 49, 52–54, 56, 59–61, 66, 71–73, 80, 83], while machined, polished, hydroxyapatite-coated, calcium-phosphate-coated, alkali-treated, and zirconia surfaces were investigated less frequently [19, 40, 42, 48, 50, 51, 55, 63–65, 70, 75–79, 81, 82, 84, 86].

**Table 2:** Characteristics of studies on CAP for osseointegration

Study type	Authors	Target population	Total sample size	Surface characteristics	CAP settings			
					**Time**	**Power**	**Gas**	**Distance**
In vivo	Wu, C., et al. (2023) [49]	Titanium implants	48	Machined smooth and sandblasted and acid-etched	90 s	NR	Argon with O_2_	45 mm
	Shon, W.J., et al. (2014) [50]	Zirconia implants	100	Smooth and roughened	60 s	NR	Helium	NR
	Zhou, X., et al. (2022) [23]	Titanium implants	20	NR	180 s	NR	Helium with 0.1% O_2_	5 mm
	Giro, G., et al. (2013) [51]	Titanium implants	24	Semicrystalline calcium-phosphate coated and moderately roughened	80 s	65 W	Argon and compressed air	NR
	Shi, Q., et al. (2015) [52]	Titanium implants	12	Sandblasted roughened	3 min	NR	Helium with 0.1% O_2_	5 mm
	Teixeira, H.S., et al. (2012) [53]	Titanium implants	36	Alumina-blasted and acid-etched	80 and 240 s	NR	Argon	NR
	Naujokat, H., et al. (2019) [54]	Titanium implants	16	Sandblasted/abrasive-blasted and acid-etched	240 s	NR	Argon	7 mm
	Krautwald, L., et al. (2022) [55]	Zirconia implants	54	Implant threads: micro-roughened Implant upper part: polished	12 min	24 W	Argon	NR
	Coelho, P.G., et al. (2012) [56]	Titanium implants	24	Alumina-blasted and acid-etched	240 s	NR	Argon	NR
	Danna, N.R., et al. (2015) [57]	Titanium implants	56	Grit-blasting/acid-etched and calcium phosphate-coated	80 s	65 W	Compressed air	NR
	Guastaldi, F.P., et al. (2013) [58]	Titanium implants	40	Sandblasted	NR	65 W	Argon	NR
	Hung, Y.-W., et al. (2018) [59]	Titanium implants	36	Sandblasted and acid-etched	60 s	NR	Argon with O_2_	NR
	Jang, M.-H., et al. (2021) [60]	Titanium implants	41	Sandblasted and acid-etched	10 min	NR	Nitrogen	3 mm
	Berger, M.B., et al. (2022) [61]	Titanium implants	72	Calcium-phosphate grit-blasted and acid-etched	30 s	NR	NR	NR
	Sandino, A.I., et al. (2025) [20]	Titanium implants	36	Alumina-blasted and acid-etched	60 s	NR	Argon	3 mm
In vitro	Karaman, O., et al. (2018) [62]	Titanium disks	NR	CAP-treated and arginine-glycine-aspartic acid peptide-coated	5, 15, 30, 45, 60, 120 and 180 s	NR	Ambient air	2 mm
	Lee, J.-H., et al. (2017) [36]	Titanium disks	NR	Roughened	10 s	NR	Nitrogen Ammonia Air	3 mm
	Lee, S.-K., et al. (2022) [42]	Zirconia disks	For osteoblast study: 90 Others: NR	Polished	For osteoblast study: 60 and 120 s Others: NR	300 W	Argon with 0.8% O_2_	NR
	Canullo, L., et al. (2017) [63]	Titanium disks	216	Machined, titanium plasma-sprayed roughened and zirconia-blasted and acid-etched	12 min	10 W	Argon	NR
	Wagner, G., et al. (2022) [64]	Titanium and zirconia disks	NR	Titanium disc: machined and ultra-roughened Zirconia disc: medium roughened	60 and 120 s	NR	Ambient air	10 mm
	Yang, Y., et al. (2018) [40]	Titanium disks	NR	Mechanically polished	2, 4 and 6 min	NR	Atmospheric air	15 mm
	Lee, H.-J., et al. (2015) [65]	Titanium disks	NR	Hydroxyapatite-coated	180 s	200 W	Argon with O_2_	NR
	Matthes, R., et al. (2017) [66]	Titanium disks	42	Sandblasted and acid-etched	600 and 1440 s	3.5 W	Argon	5 mm
	Duske, K., et al. (2012) [67]	Titanium disks	360	Machined, sandblast-etched, hydrophilic sandblast-etched, diamond-bur treated and Airflow®/air-abrasion treated	30, 60 and 120 s	2–3 W	Argon Argon with 0.2% O₂ Argon with 1.0% O₂	5 mm
	Canullo, L., et al. (2017) [68]	Titanium disks	720	Machined, titanium plasma-sprayed and zirconia-blasted and acid-etched	30, 60 and 120 s	8 W	Argon	2 mm
	Kim, I.-H., et al. (2015) [69]	Titanium disks	NR	Mechanically polished and sandblasted	180 s	200 W	Argon with O_2_	NR
	Smeets, R., et al. (2019) [70]	Zirconia disks	364	Zirconia-blasted roughened	12 min	24 W	Argon with O_2_	NR
	Duske, K., et al. (2015) [39]	Titanium disks	80	Sandblasted-etched	1080 s	2–3 W	Argon with 1% O_2_	5 mm
	Lee, H., et al. (2022) [71]	Titanium implants	NR	Sandblasted and acid-etched	60 s	NR	Ambient air	NR
	Gund, M.P., et al. (2024) [72]	Titanium disks	144	Sandblasted and acid-etched	NR	5 W	Helium with 5 sccm O_2_	2 mm
	Berger, M.B., et al. (2021) [73]	Rectangular titanium samples	NR	Grit-blasted and acid-etched	30 s	NR	Nitrogen	NR
	Wang, L., et al. (2020) [74]	Titanium disks	NR	Machined smooth and sandblasted and acid-etched	90 s	NR	Argon with 5% O_2_	20 mm
	Jha, N., et al. (2017) [75]	Zirconia disks	NR	Smooth	3, 5, 10, 20 and 30 min	NR	Nitrogen with O_2_	2 mm
	Han, I., et al. (2012) [19]	Titanium disks	NR	Smooth	2 s, 3 s, 4 min and 8 min	NR	Helium	NR
	Choi, S.-H., et al. (2016) [76]	Titanium disks	NR	Machined	10 min	NR	Compressed air	3 mm
	Martins, G.M., et al. (2024) [77]	Titanium disks	NR	Polished	NR	2.4 W	Helium	NR
	Morais, I.D., et al. (2025) [78]	Titanium disks	34	Polished	15 min	NR	Helium	10 mm
	HU, H., et al. (2021) [79]	Titanium disks	NR	Polished	30 s	NR	Active gas	5 mm
Combination of in vitro and in vivo	Dong, Y., et al. (2021) [80]	Titanium implants	In vitro: NR In vivo: 2	Sandblasted	7–75 s	< 50 W	Argon with O_2_	Entire implant submerged
	Yan, S., et al. (2022) [81]	In vitro: titanium disks In vivo: titanium implants	In vitro: NR In vivo: 24	Polished	30 s	3–10 W	Air or nitrogen	5 mm
	Zeng, Y., et al. (2020) [48]	In vitro: titanium disks In vivo: titanium implants	In vitro: NR In vivo: 16	Mechanically polished then alkali-treated	30 s	NR	NR	5 mm
	Zheng, Z., et al. (2020) [82]	In vitro: titanium disks In vivo: titanium implants	In vivo: NR In vitro: NR	Smooth/polished with a few scratches	60 s	20–50 W	Argon with O_2_	NR
	Long, L., et al. (2022) [83]	Titanium implants	In vivo: 36 In vitro: NR	In vivo: sandblasted large-grit and acid-etched; sandblasted large-grit and acid-etched and nitrogen-protected processing In vitro: sandblasted large-grit and acid-etched	30 s	20–50 W	Argon with 0.3% O_2_	NR
	Komasa, S., et al. (2021) [84]	In vitro: titanium disks In vivo: titanium implants	In vivo: 24 In vitro: NR	Smooth	30 s	NR	Argon	10 mm
	Zheng, Z., et al. (2022) [85]	In vitro: titanium disks In vivo: titanium implants	NR	NR	60 s	NR	Argon with 0.5% O_2_	NR
	Tsujita, H., et al. (2021) [86]	In vitro: titanium disks In vivo: titanium implants	In vitro: NR In vivo: 20	Machined smooth	30 s	NR	NR	10 mm

CAP treatment parameters varied considerably across studies. Treatment duration ranged from 2 s to 30 min [19, 75], although exposure times between 30 and 240 s were most commonly reported [20, 23, 42, 48–54, 56, 57, 59, 61, 64, 65, 67–69, 71, 73, 74, 79, 81–86]. Argon was the predominant working gas, either alone or combined with oxygen, and was used in most studies [20, 23, 39, 42, 48, 49, 53–56, 58, 59, 63, 65–70, 74, 80, 82–85] whereas helium-, nitrogen-, air-, and compressed-air-based plasma systems were evaluated less frequently. The treatment distance ranged from direct contact to 45 mm, with distances of 2–10 mm being most commonly applied [20, 23, 36, 39, 48, 52, 54, 60, 62, 64, 66–68, 72, 75, 76, 78, 79, 81, 84, 86]. Overall, the evidence was primarily derived from studies using argon-based CAP on titanium sandblasted and acid-etched or roughened surfaces, whereas investigations involving zirconia implants, direct comparisons among plasma sources, and standardized CAP treatment protocols remain limited.

## Discussion

**Fig. 2:**
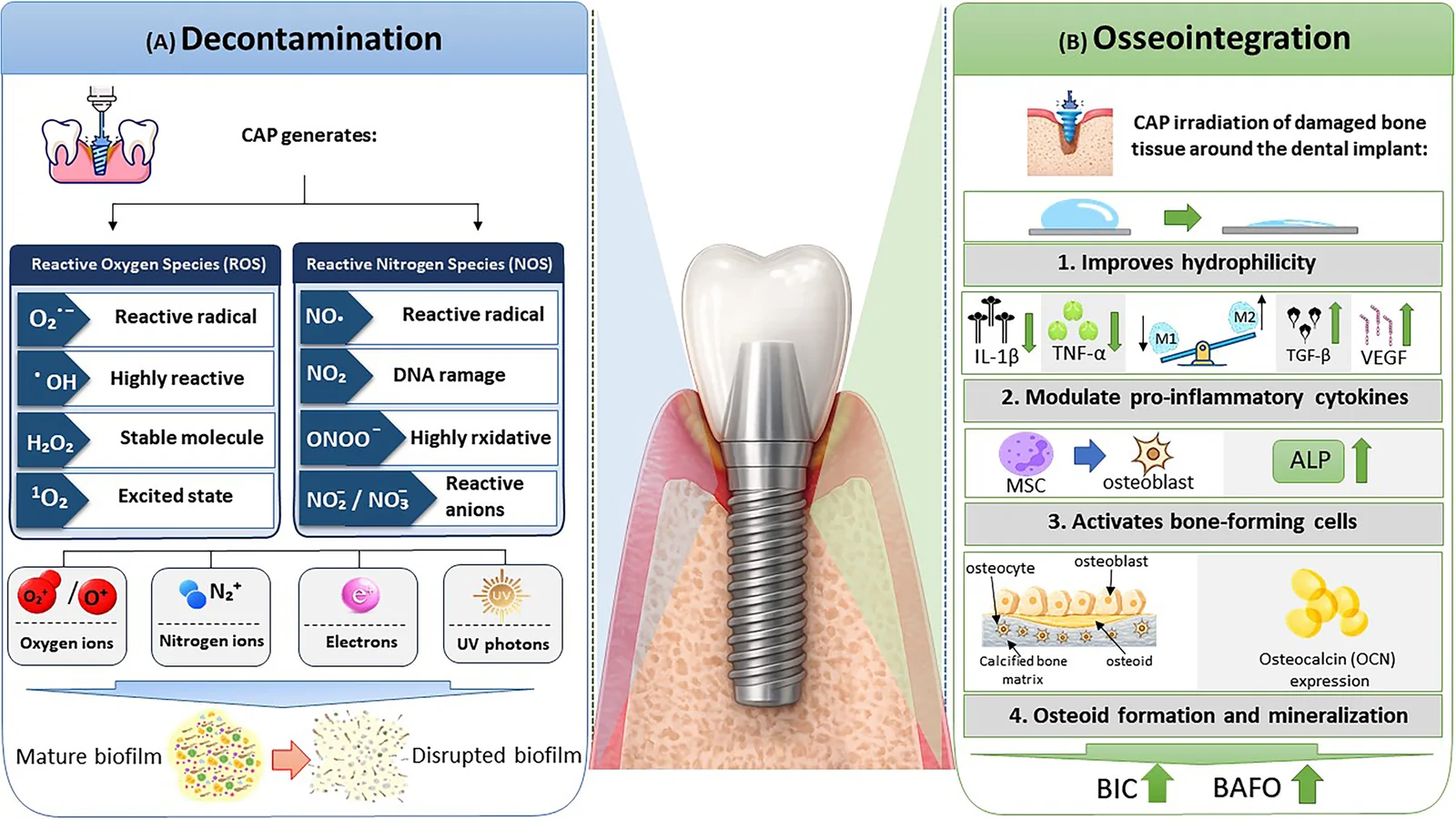
Schematic illustration of the potential biological effects of CAP on implant surface decontamination and osseointegration-related processes

Peri-implantitis remains a persistent clinical challenge, largely due to the difficulties associated with both its prevention and management. CAP has been successfully applied across a broad spectrum of medical disciplines, including dermatology, oncology, ophthalmology, wound healing, tissue engineering, and antimicrobial therapy [87–92]. In the field of dentistry, CAP has been explored for sterilization, caries management, biofilm control, disinfection, adhesive restoration, tooth whitening, and soft tissue healing [93]. Recently, CAP has attracted increasing attention for peri-implantitis management due to its potential to decontaminate implant surfaces and support biological processes associated with osseointegration. Based on the current literature, Fig. 2 provides a schematic overview of the potential biological effects of CAP on implant surface decontamination and osseointegration-related processes. Within this context, the present findings suggest that CAP demonstrates promising biological effects in preclinical settings. However, as the current evidence is predominantly derived from in vitro, ex vivo, and animal studies, its direct clinical applicability remains limited.

### Effect of CAP on peri-implantitis management: decontamination

In vitro studies consistently demonstrate that CAP and related plasma-based technologies exert potent antibacterial effects, although the magnitude of efficacy varies with treatment parameters and exposure duration. Beyond the reduction of bacterial load, CAP has been reported to induce alterations in biofilm morphology and implant surface characteristics, suggesting that plasma treatment not only achieves microbial decontamination but may also modify the implant surface in ways that favor subsequent tissue integration [29, 34, 43].

Peri-implant infections are typically driven by complex multispecies biofilms, which exhibit enhanced resistance to conventional decontamination strategies and pose significant clinical challenges [37]. Panariello et al. [18] evaluated the efficacy of low-temperature plasma against multispecies peri-implant biofilms composed of *Actinomyces naeslundii, Porphyromonas gingivalis, Streptococcus oralis, and Veillonella dispar*. They reported that treatment for 1–5 min significantly reduced bacterial counts, suggesting substantial antibacterial activity of CAP against complex microbial communities under experimental conditions. Complementary studies by Jungbauer et al. [35] further revealed that CAP exerts a dual mode of action, achieving rapid biofilm decontamination while simultaneously inducing surface modifications that may contribute to the long-term prevention of bacterial recolonization.

Across the included studies, CAP generally demonstrated antibacterial activity across a range of peri-implant pathogens and biofilm models. This effect is generally attributed to the generation of reactive oxygen and nitrogen species, which can disrupt microbial cell membranes and metabolic pathways. However, it should be noted that most studies were conducted under controlled laboratory conditions using simplified biofilm models, which may not fully replicate the structural complexity and resilience of biofilms found in clinical peri-implantitis. Therefore, while the antimicrobial potential of CAP appears robust at the experimental level, its effectiveness in clinical scenarios remains to be validated.

Although titanium remains the most frequently studied substrate, CAP treatment has also been applied to zirconia [17, 24, 42]. These investigations suggested that CAP may reduce bacterial colonization on zirconia surfaces and may improve surface conditions associated with host cell attachment and activity. Moreover, treatment parameters, including plasma exposure time, power, and distance, modulate both direct antimicrobial effects and indirect cellular responses, highlighting the importance of optimizing CAP protocols to achieve balanced biological outcomes.

While in vitro studies have established the antibacterial and biofunctional potential of CAP, in vivo and ex vivo investigations are critical to assess its translational relevance for implant surface decontamination and peri-implantitis management. Evidence from in vivo and ex vivo studies suggested that CAP treatment may reduce markers of peri-implant inflammation [20, 22, 23, 48]. Moreover, ex vivo investigations demonstrated that CAP was associated with reductions in biofilm load on titanium implant surfaces, even under the complex conditions of in situ biofilm development [45–47]. CAP efficacy is influenced by biofilm maturity and treatment parameters. Younger biofilms are more susceptible to plasma-induced disruption, whereas mature biofilms (typically ≥ 72 h) exhibit greater resistance, necessitating longer exposure times or adjunctive treatments for comparable decontamination [45, 46]. Importantly, studies have shown that CAP exhibits selective antimicrobial effects while maintaining the microstructure of titanium surfaces, a critical factor in preserving the physicochemical and biological integrity of implants.

Across the included preclinical studies, the combination of CAP with mechanical debridement was generally associated with greater reductions in biofilm burden than either modality alone. Combined protocols have been reported to enhance biofilm removal and reduce bacterial regrowth and adherence under experimental conditions [26, 28, 41]. CAP has also demonstrated improved antifungal activity against *Candida albicans*, a pathogen implicated in denture stomatitis and peri-implant infections [32], achieving greater efficacy than conventional chemical disinfectants such as chlorhexidine and sodium hypochlorite, likely due to its ability to penetrate deeper into biofilm structures. The enhanced efficacy of combined mechanical and CAP treatment likely reflects complementary mechanisms of action. Mechanical debridement disrupts and removes the bulk of the biofilm, whereas CAP serves as a secondary disinfectant, eradicating residual microorganisms, inhibiting biofilm regrowth, and potentially improving biological compatibility of the treated surface. This synergistic approach underscores CAP’s potential as an adjunctive modality for comprehensive implant surface decontamination and peri-implant disease management.

Overall, CAP shows potential as an adjunctive strategy for implant surface decontamination in peri-implantitis management, particularly when combined with mechanical debridement. However, given the predominance of heterogeneous preclinical evidence, its clinical effectiveness remains to be established.

### Effect of CAP on peri-implantitis management: osseointegration

CAP has been reported to influence biological processes related to osseointegration, including osteoblast attachment, cellular activity, and biochemical markers [36, 69, 72, 73]. CAP treatment enhances alkaline phosphatase expression, a critical marker of osteoblast differentiation and early bone mineralization, which may support early osseointegration-related processes [36]. Additionally, CAP increases osteocalcin levels, indicative of bone matrix formation and mineralization, suggesting a potential role in bone regeneration [82, 85]. CAP has also been reported to modulate pro-inflammatory cytokines, potentially modulating inflammatory responses relevant to peri-implantitis and supporting tissue healing [52]. Collectively, these findings suggest a potential influence on osseointegration-related parameters, although their implications for implant stability in clinical settings remain uncertain. Both in vitro and in vivo studies suggest that CAP may modify implant surface properties and influence the local biological microenvironment in ways that could support osseointegration-related processes. However, whether these findings translate into improved clinical outcomes in patients with peri-implantitis remains unknown.

In addition to its antimicrobial properties, CAP has been shown to significantly modify surface properties, particularly by increasing surface hydrophilicity [67, 70, 80]. These surface modifications have been associated with osteoblast adhesion, proliferation, differentiation, and increased BIC in preclinical models [82]. Nevertheless, these findings should be interpreted with caution, as most evidence is derived from in vitro and animal studies with relatively short observation periods and controlled experimental conditions. Therefore, the clinical relevance of these improvements in terms of long-term implant stability and osseointegration outcomes remains to be fully established.

In vitro investigations have demonstrated that CAP enhances osseointegration by modifying titanium surfaces [40, 65, 69]. Han et al. [19] reported that CAP treatment generated super-hydrophilic titanium surfaces, evidenced by a six-fold increase in surface energy, which promoted osteoblast attachment and spreading. Moreover, they reported enhanced fibronectin adsorption on CAP-treated surfaces, which further facilitated cellular adhesion and integration at the implant-tissue interface. Similarly, Sandino et al. [20] demonstrated that CAP altered titanium surface characteristics, leading to increased cell attachment and osteogenic differentiation, effects attributed to elevated surface energy and reactive oxygen species generation. These modifications were associated with reduced surface carbon content and increased hydrophilicity, creating a more favorable environment for osseointegration.

The beneficial effects of CAP are not limited to titanium surfaces. Shon et al. [50] showed that helium-based CAP treatment improved zirconia surface hydrophilicity and osseointegration without significantly altering microtopography. Giro et al. [51] observed BIC exceeding 100% and BAFO increases over 82% at 3 weeks on calcium-phosphate-coated implants treated with argon plasma. In a canine model, Coelho et al. [56] reported BIC increases exceeding 300% and BAFO improvements over 30% on rough titanium surfaces following argon plasma treatment. These findings suggest that CAP may modify surface properties, primarily by enhancing wettability, a factor commonly associated with osseointegration. Consistently, Zheng et al. [82] demonstrated that plasma treatment increased surface oxygen content, cell attachment, osteogenic differentiation, and osseointegration. Duske et al. [67] further confirmed that plasma-induced wettability consistently enhanced early cellular responses across different surface topographies, including machined, sandblasted and acid-etched, and sandblasted and acid-etched and nitrogen-protected surfaces.

Several studies have highlighted CAP’s capacity to modulate the peri-implant inflammatory environment. Zhou et al. [23] demonstrated that a modified CAP protocol combined with mechanical debridement produced significant clinical improvements in sulcus bleeding index, probing depth, and bone height compared with controls. This treatment was associated with marked reductions in pro-inflammatory cytokines such as interleukin-1β and interleukin-17. Histological analyses further revealed enhanced osseointegration and increased new bone formation, suggesting that CAP contributes to the re-establishment of a stable osseointegration interface through cytokine regulation.

The osteogenic benefits of CAP extend beyond osteoblasts. Wagner et al. [64] reported enhanced fibroblast adhesion following CAP treatment, indicating potential improvements in soft tissue integration, which may reinforce biomechanical fixation by both bone and connective tissue cells. The synergistic enhancement of these cellular responses may contribute to the formation of a favorable biological interface at the implant surface. Jang et al. [60] observed that non-thermal atmospheric pressure plasma jet-treated titanium implants exhibited increased bone volume and BIC at 4 weeks post-implantation, though the effect diminished by 8 weeks, suggesting that CAP exerts its greatest influence during early healing. Teixeira et al. [53] similarly demonstrated that brief plasma exposure (20–60 s) increased initial surface energy and improved biomechanical fixation, emphasizing the importance of CAP in the early stages of osseointegration.

In summary, CAP demonstrates potential to influence osseointegration-related processes through surface modification, cellular response enhancement, and inflammatory regulation. While these findings suggest a possible role for CAP in supporting peri-implant tissue integration, they are largely based on preclinical evidence and should be interpreted with caution. Further well-designed in vivo studies and clinical trials are required to clarify its clinical relevance.

### Factors influencing the effects of CAP

The variability observed across studies on CAP can be attributed to multiple interacting factors, including plasma treatment parameters, implant material characteristics, and differences in experimental models and plasma delivery systems. Several studies have reported that longer treatment durations and the use of argon-based plasma generally produce more pronounced improvements in surface properties, particularly by enhancing surface hydrophilicity, compared with shorter exposures or air plasma applications [20, 84]. Argon-based plasma has been associated with increased oxygen incorporation and hydrophilicity relative to air plasma or other gas compositions. Conversely, Smeets et al. [70] reported that oxygen-based CAP exhibited superior biological performance compared with argon plasma, particularly in promoting cell proliferation on zirconia surfaces. These findings suggest that variations in plasma gas composition may partly explain the discrepancies in biological outcomes and osseointegration efficacy reported across studies.

The implant material itself is another critical determinant of the response to plasma treatment. Zirconia implants display distinct surface and biological responses compared with titanium following plasma exposure. For example, the formation of surface nanoparticles on zirconia varies according to the plasma gas used, such as nitrogen versus oxygen, resulting in different surface chemistries and potentially divergent biological interactions [75]. Consequently, outcomes observed for zirconia implants cannot be directly extrapolated to titanium-based systems. Baseline surface characteristics also modulate the response to CAP; roughened titanium surfaces generally demonstrate superior outcomes compared with calcium phosphate-coated implants, likely reflecting differences in surface topography, chemical composition, and interactions with plasma-generated reactive species [56, 57].

Gas chemistry is another key variable, as different plasma gases generate distinct reactive species that influence surface modifications. Air plasma typically produces reactive oxygen and nitrogen species, ultraviolet radiation, and ions, collectively contributing to changes in surface roughness [40]. In contrast, plasmas generated from inert or mixed gases, such as argon/oxygen or helium, primarily function to remove surface carbon contaminants and introduce hydroxyl groups, enhancing surface cleanliness and hydrophilicity without substantially altering topography [19]. The configuration of the plasma system also affects treatment outcomes. Coaxial dielectric barrier discharge systems provide a more homogeneous distribution of reactive species across the implant surface, allowing effective surface modification within shorter exposure times (e.g., 30 s) without inducing thermal damage. Jet-based plasma systems, by contrast, often require longer exposure durations to achieve comparable surface effects, which may inadvertently modify surface topography [40, 74, 80].

Most studies indicate that the biological effects of plasma treatment are most pronounced during the early stages of healing, particularly for titanium implants. These effects tend to plateau over time, reflecting the initial enhancement of osseointegration mediated by improved surface characteristics and early cell attachment. As bone remodeling progresses, the influence of plasma-induced surface modifications diminishes, suggesting that the primary advantage of CAP lies in promoting the initial phase of implant-tissue integration.

Recent systematic reviews have addressed specific aspects of CAP in implant dentistry. Barausse et al. [94] focused on osseointegration, highlighting improvements in surface properties and early bone healing, while Alqutaibi et al. [95] evaluated antibacterial efficacy, demonstrating reductions in bacterial load. In contrast, the present scoping review integrates these domains by examining both decontamination and osseointegration. This approach reflects the multifactorial nature of peri-implantitis and provides a more comprehensive, conceptually integrated overview of CAP-related effects.

### Limitations of the study

A primary limitation of this review is that the literature search was confined to four electronic databases, which may have led to the omission of relevant studies. In addition, only English-language publications were included due to constraints related to resources, time, and translation, which might have resulted in the underrepresentation of studies published in other languages. Although the inclusion of peer-reviewed articles enhanced methodological rigor and data reliability, future reviews should consider incorporating multilingual sources to achieve a more comprehensive and globally representative synthesis. Another important limitation relates to the nature of the included evidence. The studies were highly heterogeneous in terms of materials, plasma devices, gas composition, exposure parameters, biological models, and outcome measures. While expected in a scoping review, this variability limits direct comparability and precludes firm conclusions regarding optimal CAP parameters or standardized protocols. Moreover, as most studies were preclinical and did not consistently reproduce a true peri-implantitis condition, the direct clinical applicability of the findings remains limited.

## Conclusions

Within the limitations of the available evidence, CAP has demonstrated potential to support implant surface decontamination and osseointegration-related processes in preclinical settings, addressing two fundamental biological processes involved in peri-implantitis. However, the included studies were highly heterogeneous and did not consistently reproduce a true peri-implantitis model, which limits direct clinical interpretation. Rather than providing definitive clinical evidence, the present findings support the biological plausibility of CAP as a potential adjunctive approach in peri-implantitis management. Future research should focus on standardized experimental models, detailed reporting of plasma parameters, and well-designed clinical trials to clarify its clinical effectiveness and optimize treatment protocols.

## Supplementary Information

Supplementary Material 1.

Supplementary Material 2.

## Data Availability

The authors confirm that the data supporting the findings of this study are available within the article.

## Abbreviations

CAP: Cold Atmospheric Plasma
PRISMA-ScR: Preferred Reporting Items for Systematic Reviews and Meta-Analyses Extension for Scoping Reviews
PCC: Population, Concept, Context
CFU: Colony-Forming Units
BIC: Bone-to-Implant Contact
BAFO: Bone Area Fraction Occupancy
