# Supplementary material for: Cold atmospheric plasma for implant surface decontamination and osseointegration: a scoping review with implications for peri-implantitis management
Source: BMC Oral Health. 2026 Jul 17;26:1983. doi: 10.1186/s12903-026-09164-y (PMC13656613; doi:10.1186/s12903-026-09164-y)
Supplement: Supplementary file 2 [file 12903_2026_9164_MOESM2_ESM.docx]

Detailed search strategies for all databases

Search period: January 2010 – July 2025

Language restriction: English

Search date: July 2025

1. PubMed

Search query:

((“Cold Atmospheric Plasma”[tiab] OR “CAP”[tiab] OR “cold plasma”[tiab] OR “nonthermal plasma”[tiab] OR “NTP”[tiab] OR “nonthermal atmospheric plasma”[tiab] OR “NTAPP”[tiab] OR “cold atmospheric pressure plasma”[tiab] OR “CAPP”[tiab] OR “argon plasma”[tiab] OR “helium plasma”[tiab] OR “oxygen plasma”[tiab] OR “nitrogen plasma”[tiab] OR “air plasma”[tiab] OR “plasma gases”[tiab] OR “plasma jet”[tiab] OR “dielectric barrier discharge”[tiab]) AND (“Peri-Implantitis”[MeSH Terms] OR “peri-implantitis”[tiab] OR “dental implant”[tiab] OR “oral implant”[tiab] OR “oral implantology”[tiab] OR “titanium implant”[tiab] OR “zirconia implant”[tiab] OR “implantology”[tiab])) NOT (review[pt] OR editorial[pt] OR comment[pt] OR case reports[pt])

Filters applied: • Publication years: 2010–2025 • Language: English • Full-text available

Records retrieved: n = 163

2. Scopus

Search query:

TITLE-ABS-KEY ( “cold atmospheric plasma” OR CAP OR “cold plasma” OR “nonthermal plasma” OR “non-thermal plasma” OR “nonthermal atmospheric plasma” OR “non-thermal atmospheric plasma” OR NTAPP OR “cold atmospheric pressure plasma” OR “plasma jet” OR “dielectric barrier discharge” )

AND

TITLE-ABS-KEY ( “peri-implantitis” OR periimplantitis OR “peri-implant disease” OR “peri-implant diseases” OR ( ( “dental implant” OR “dental implants” OR “titanium implant” OR “titanium implants” OR “zirconia implant” OR “zirconia implants” ) W/5 ( biofilm OR biofilms OR decontamination OR disinfect* OR steriliz* OR antimicrobial OR antibacterial OR osseointegration OR osteoblast OR osteoblasts ) ) )

AND NOT ( DOCTYPE(re) OR DOCTYPE(ed) OR DOCTYPE(le) OR TITLE-ABS-KEY(“case report” OR “case reports” OR comment OR commentary) )

AND LIMIT-TO(LANGUAGE, “English”)

Filters applied: • Publication years: 2010–2025 • Language: English

Records retrieved: n = 114

3. Web of Science

Search query:

TS= ( “cold atmospheric plasma” OR CAP OR “cold plasma” OR “nonthermal plasma” OR “non-thermal plasma” OR “nonthermal atmospheric plasma” OR “non-thermal atmospheric plasma” OR NTAPP OR “cold atmospheric pressure plasma” OR “plasma jet” OR “dielectric barrier discharge” )

AND

TS= ( “peri-implantitis” OR periimplantitis OR “peri-implant disease” OR “peri-implant diseases” OR ( ( “dental implant” OR “dental implants” OR “titanium implant” OR “titanium implants” OR “zirconia implant” OR “zirconia implants” ) NEAR/5 ( biofilm OR biofilms OR decontamination OR disinfect* OR steriliz* OR antimicrobial OR antibacterial OR osseointegration OR osteoblast OR osteoblasts OR hydrophilicity ) ) )

NOT

DT= ( Review OR Editorial Material OR Letter OR Meeting Abstract )

Filters applied: • Publication years: 2010–2025 • Language: English

Records retrieved: n = 103

4. Google Scholar

Search query:

(“cold atmospheric plasma” OR “cold plasma” OR “nonthermal plasma” OR “cold atmospheric pressure plasma”)

AND

(“peri-implantitis” OR “dental implant”)

AND

( biofilm OR decontamination OR osseointegration OR osteoblast OR osteoblasts OR hydrophilicity )

NOT

DT= ( Review OR Case Report)

Filters applied: • Publication years: 2010–2025 • Language: English

Records retrieved: n = 70

Summary of Search Results

PubMed: 163 records

Scopus: 114 records

Web of Science: 103 records

Google Scholar: 70 records

Total records identified: 450
